# Sound category habituation requires task-relevant attention

**DOI:** 10.3389/fnins.2023.1228506

**Published:** 2023-10-24

**Authors:** Howard S. Moskowitz, Elyse S. Sussman

**Affiliations:** ^1^Department of Otorhinolaryngology-Head and Neck Surgery, Albert Einstein College of Medicine, Bronx, NY, United States; ^2^Department of Neuroscience, Albert Einstein College of Medicine, Bronx, NY, Unites States

**Keywords:** categorical perception, attention, speech, implicit learning, event-related brain potentials (ERPs)

## Abstract

**Introduction:**

Processing the wealth of sensory information from the surrounding environment is a vital human function with the potential to develop learning, advance social interactions, and promote safety and well-being.

**Methods:**

To elucidate underlying processes governing these activities we measured neurophysiological responses to patterned stimulus sequences during a sound categorization task to evaluate attention effects on implicit learning, sound categorization, and speech perception. Using a unique experimental design, we uncoupled conceptual categorical effects from stimulus-specific effects by presenting categorical stimulus tokens that did not physically repeat.

**Results:**

We found effects of implicit learning, categorical habituation, and a speech perception bias when the sounds were attended, and the listeners performed a categorization task (task-relevant). In contrast, there was no evidence of a speech perception bias, implicit learning of the structured sound sequence, or repetition suppression to repeated within-category sounds (no categorical habituation) when participants passively listened to the sounds and watched a silent closed-captioned video (task-irrelevant). No indication of category perception was demonstrated in the scalp-recorded brain components when participants were watching a movie and had no task with the sounds.

**Discussion:**

These results demonstrate that attention is required to maintain category identification and expectations induced by a structured sequence when the conceptual information must be extracted from stimuli that are acoustically distinct. Taken together, these striking attention effects support the theoretical view that top-down control is required to initiate expectations for higher level cognitive processing.

## 1. Introduction

In the modern-day world we are constantly inundated by a cavalcade of sensory input from our surrounding environment. Our brains must process this information efficiently to facilitate appropriate reactions and responses. The ability to perceive and monitor sounds to which we are not specifically attending serves an important role for general functioning and safety. However, a gate or mechanism must exist to govern this important function. How and when we make the decision regarding which sounds meet a sufficient level of importance would promote welfare while also controlling utilization of important cognitive and behavioral functions that could be directed elsewhere.

The exact manner by which these processes are generated continues to be a source of research and controversy. The classical view of perception maintains a feedforward view: information is received from the environment, processed at higher brain levels and then a response to the input is generated (Mumford, 1992). Any mismatch with the actual sensory input constantly updates the prediction based on this error signal (Rao and Ballard, 1999). An alternative concept suggests that we predict the nature of incoming sensory information based on previous experiences, to process efficiently and to allocate resources to novel stimuli (Friston, 2005). The concept governing this process is explained by predictive processing theories that suggest that the brain generates models that automatically anticipate and predict upcoming sensory input based on the recent history of the sensory input (Clark, 2013). A predictive model is generated in higher cortical areas and is communicated through feedback connections to lower sensory areas (Rao and Ballard, 1999; Friston, 2005). Recently, the concept of predictive processing has been validated by several brain imaging studies investigating predictive feedback and the processing of prediction errors (den Ouden et al., 2009; Friston and Kiebel, 2009; Egner et al., 2010; Jiang et al., 2013; Alink and Blank, 2021). However, these models do not take into account the precise nature of the stimulus input on which the predictions are based, or how they are established or maintained in memory. Accordingly, these issues are still being debated (Walsh et al., 2020). There is an essential lack of understanding of (1) the role attention plays in forming the predictions themselves; and (2) how the predictions are instantiated, such as whether they are based on simple stimulus repetition, attentional control, or something else (Rao and Ballard, 1999; Friston et al., 2006; Summerfield et al., 2008; Bubic et al., 2010; Larsson and Smith, 2012; Clark, 2013; Walsh et al., 2020).

**Figure 1:**
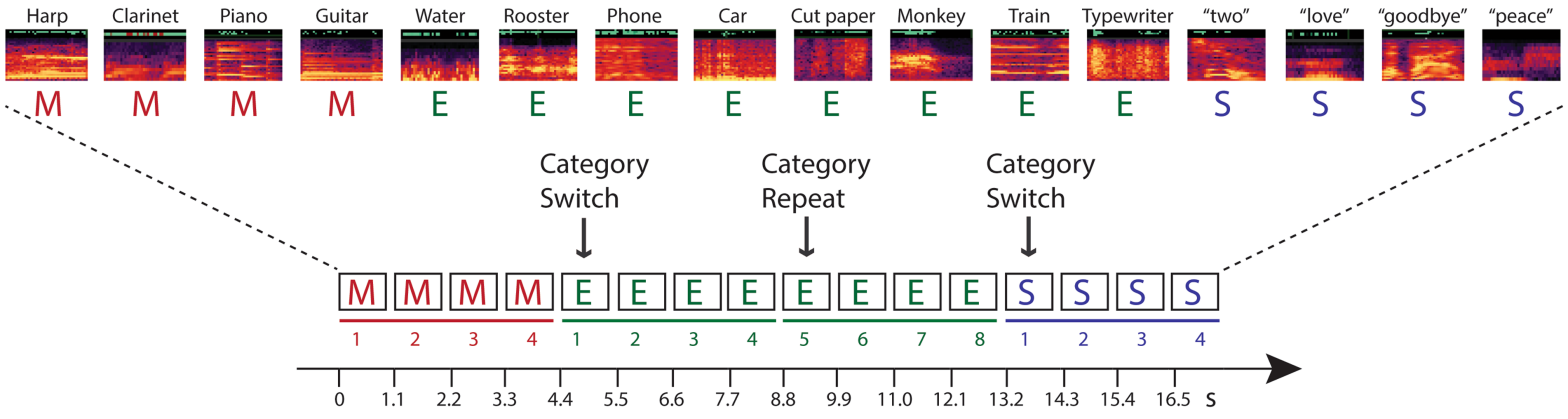
Schematic of the experimental paradigm. Sounds from three categories – music (M, red font), environment (E, green font), and speech (S, blue font) – were presented in categorical groups of four stimuli (denoted by solid line in their respective color, and numbered 1–4). Randomly, the category switched to a new category (indicated with an arrow and labeled ‘Category Switch’) or the category repeated (indicated with an arrow and labeled ‘Category Repeat’). When the category randomly repeated, eight successive tokens from the same category were presented (denoted by a solid line and numbered 5–8 for the repeated category). A random sample of the sounds from each category are depicted with their respective spectrograms, and labeled above (see Appendix I for full list of the sounds). Each within-category token was unique in every four-token group. Stimuli were presented isochronously, one sound every 1.1 s, in each stimulus block, with no demarcation of the switch or repeat trials.

The current study tests hypotheses that investigate these questions within predictive processing theories. Namely, we designed a study that dissociated stimulus-specific adaptation from semantic categorical repetition to determine whether higher-level, conceptual expectations can be encoded from stimulus repetition when the stimulus repetition is based on category membership and there is no repetition in the acoustic characteristics of the sound tokens. To do this, we implemented a novel paradigm for measuring repetition suppression to assess the reduction of neural activity to repeated stimuli (Moskowitz et al., 2020). Participants heard sounds presented in groups consisting of stimuli by semantic category (spoken words, sounds of musical instruments, environmental sounds) (Figure 1). Each four-stimulus category group was randomly followed by another group of four sounds that were from a different sound category (switch) or from the same category (repeat). There were no repeated stimulus tokens: No sounds were physical repeats of any other sound in the stimulus blocks, only the category was repeated or switched. Thus, a change in response induced by repetition of the category could not be specifically due to token-specific sensory adaptation or to repetition suppression.

We used event-related brain potentials during passive auditory and active auditory listening conditions to measure the brain’s response to the same categorical sounds when the categorical aspect of the sounds was relevant compared to when the categorical aspect of the task was irrelevant. The P3a component reflects involuntary orienting to a salient sound regardless of the direction of attention (Friedman et al., 2001; Polich, 2007; Fonken et al., 2020), with its greatest amplitude at frontocentral locations (e.g., Cz electrode). The P3b component is a non-modality-specific index of task-related processing (Sutton et al., 1965; Picton, 1992) and generally has its largest amplitude over parietal scalp electrodes (e.g., Pz) (Polich, 2007). The P3b is elicited when attention is focused on stimuli to identify a target (Polich and Criado, 2006). Therefore, it is elicited by task-relevant but not task-irrelevant stimuli, and can reflect category perception (Maiste et al., 1995). The different neural substrates of the P3a (at Cz) and P3b (at Pz) components reflect different aspects of attention (attentional orienting and target detection, respectively) (Fonken et al., 2020). The sensory-specific N1 component is an obligatory response of the ERPs, elicited by sound onsets and its amplitude is reduced with stimulus repetition (Näätänen and Picton, 1987; Budd et al., 1998; Hsu et al., 2016; Rosburg and Mager, 2021) and increased with focused attention (Hillyard et al., 1998). These primary dependent measures, the P3a, P3b, and N1 components, provided neural responses to the categorical sounds that indexed involuntary orienting to sounds during passive and active auditory task listening (P3a) observable at the Cz electrode, an index of task performance during active auditory task listening (P3b) observable at the Pz electrode, and an index of auditory-specific sensory process (N1) elicited during passive and active auditory tasks, observable at Cz.

In this approach, we measured the effects of conceptual “repetition suppression” to sound categories and not to individual repeating physical stimuli. Further, responses to the sounds when they were task-relevant were compared with responses to the sounds when they were task-irrelevant to further test the automaticity of conceptual category representation – to evaluate the role of directed attention in maintaining predictions.

Our results demonstrated conceptual categorical expectation, implicit learning, and a speech perception bias only when the sounds were attended, and a sound categorization task was performed with them. These results indicate that attentional control is required to maintain semantic category identification, and that higher-level processes use that information to predict upcoming events.

## 2. Materials and methods

### 2.1. Participants

Ten adults ranging in age from 19–37 years (mean = 28.5, SD = 5.5) were paid to participate in the study. All participants passed a hearing screening at 20 dB HL or better at 500, 1,000, 2,000, and 4,000 Hz in the left and right ears and had no reported history of neurological or otologic disorders. Procedures were approved by the Institutional Review Board of the Albert Einstein College of Medicine (Bronx, NY) where the study was conducted. The examiner described the procedure to all participants in accordance with the Declaration of Helsinki who subsequently gave written consent and were paid for their participation.

Results of a power analysis conducted using Statistica software (Tibco), with a two-tailed *t*-test for dependent means, a medium effect size (*d* = 0.50) and an alpha of 0.05. determined that a sample size of nine participants would yield power of 0.90 to detect differences. The total of ten participants included in the current study thus exceeds the number required to obtain sufficient statistical power.

### 2.2. Stimuli

Stimuli were naturally produced complex sounds (32-bit stereo; 44,100 Hz digitization) obtained from free online libraries (Appendix I). Three categories of sounds were presented, of which there were 25 different tokens of spoken speech, 25 different tokens of musical instruments, and 57 different tokens of various environmental sounds. Speech sounds were naturally spoken words (e.g., “hello,” “goodbye”); music sounds were taken from various musical instruments (e.g., piano, flute, bass); and environmental sounds were taken from a range of sources, including nature (e.g., water dripping), vehicles (e.g., engine revving), household (e.g., phone ring), and animals (e.g., bird chirp). We modified all the sounds to be 500 ms in duration, with an envelope rise and fall times of 7.5 ms at onset and offset using Adobe Audition software (Adobe Systems, San Jose, CA). To verify that 500 ms in sound duration was sufficient to identify and distinguish the sound categories (e.g., spoken word, instrumental, environmental), three lab members (who were not included in the study) categorized a set of 150 sounds. The final set of 107 sounds used in the study had unanimous agreement as belonging to a category of speech, music, or environment. All 107 stimuli were equated for loudness using the root mean square (RMS) amplitude with Adobe Audition software. Categorical sounds were calibrated with a sound pressure level meter in free field (Brüel and Kajaer, Denmark) and presented through speakers at 65 dB SPL with a stimulus onset asynchrony (SOA) of 1.1 s.

### 2.3. Procedures

Participants sat in a comfortable chair in an electrically shielded and sound-controlled booth (IAC Acoustics, Bronx, NY). Stimuli were presented via two speakers placed approximately 1.5 m, 45° to the left of center and 1.5 m, 45° to the right of center from the seated listener.

There were two task conditions: *passive auditory* and *active auditory*. During the *passive auditory* condition, participants had no specific task with the sounds. They watched a captioned silent movie of their choosing during the presentation of the sounds. The experimenter monitored the EEG to ensure that participants were reading the closed captions. In the *active auditory* condition, participants listened to the sounds and performed a three-alternative forced-choice task. Participants were instructed to listen to and classify each sequential sound by pressing one of three buttons labeled on a response keypad that uniquely corresponded to the sound category (speech, music, or environment). Participants were not provided with any information about the patterned structure of the stimulus sequence at any time. Thus, the patterned structure could be extracted by implicit learning regardless of the condition in which they were presented.

A total of 3,840 stimuli were presented in 16 separately randomized stimulus blocks (240 stimuli per block), eight stimulus blocks per condition. Stimuli were presented in continuous sequences of 240 stimuli, patterned by categorical groups of four stimuli (spoken words, musical instruments, environmental sounds), with an equal distribution of the categories in each condition (0.33 speech, 0.33 music, and 0.33 environmental). Category switches and repeats occurred randomly within each stimulus block. There were no repeated stimuli within any of the stimulus groups. Every sound token was unique in each categorical group (e.g., the sounds of the instruments harp, piano, clarinet, and guitar could be repetitions in the category of the music group, Figure 1). Categories switched randomly after four stimuli 70% of the time overall (336 switch trials per condition), and randomly repeated categories after four stimuli 30% of the time (144 repeat trials per condition). Presentation of sound groups was quasi-randomized such that categories could only repeat one time. Thus, sounds occurred in groups of either four or eight repetitions of any category. Participants were not informed about the structure of the sequences at any time and there was no demarcation to indicate when the category switched, or when the category repeated within a stimulus block; sound tokens were presented isochronously throughout every stimulus block. Thus, position #1 stimuli were only a ‘first position’ stimulus based only with implicit detection of the patterned categorical grouping.

Task conditions were randomized across participants, with half of participants presented with the passive condition first and half presented with the active condition first. Recording time was approximately 35 min per condition, with a snack break at the halfway point at which time the participant was unhooked from the amplifiers and could walk around. Total session time including cap placement, recording time, and breaks was approximately 2 h.

### 2.4. Electroencephalogram recordings

A 32-channel electrode cap incorporating a subset of the International 10–20 system was used to obtain EEG recordings. Additional electrodes were placed over the left and right mastoids (LM and RM, respectively). An external electrode placed at the tip of the nose was used as the reference electrode. Horizontal electro-oculogram (EOG) was monitored with the F7 and F8 electrode sites and vertical EOG was monitored using a bipolar configuration between FP1 and an external electrode placed below the left eye. Impedances were kept below 5 kΩ at all electrodes throughout the recording session. The EEG and EOG were digitized (Neuroscan Synamps amplifier, Compumedics Corp., Texas, United States) at a sampling rate of 1,000 Hz (0.05–200 Hz bandpass). EEG was filtered off-line with a lowpass of 30 Hz (zero phase shift, 24 dB rolloff).

### 2.5. Data analysis

This report includes data from all 10 participants in the study. There were no exclusions.

*Behavioral Data*: Hit rate (HR) and reaction time (RT) were calculated for the responses to each of the sounds, separately by category (speech, music, and environment and by stimulus position (1–8)). Hits were counted when responses occurred 100–1,100 ms from the onset of the stimulus. The mean HR was calculated as the total number of correctly identified stimuli divided by the number of stimuli in each category for each position. RT was calculated for each sound from sound onset. Means were derived for each stimulus category, in each position separately.

*ERP Data*: The filtered EEG was segmented into 4,500 ms epochs, starting from 200 ms pre-stimulus and ending 4,300 ms post-stimulus onset from position 1 for the switch category to display ERP responses consecutively in positions 1–4, and from the onset of position 5 for the repeat category to display ERP responses consecutively in positions 5–8. Due to the length of these epochs, ocular artifact reduction was performed on all participants using Neuroscan EDIT software. This Singular Value Decomposition transform method is used to identify the blink component. From the continuous EEG, a file was created that reflected the spatial distribution of the blink and then used to remove the blink. The blink-corrected data were then baseline-corrected across the whole epoch (the mean was subtracted at each point across the epoch). After baseline correction, artifact rejection criteria were set to ±75 mV. On average, 89% of all trials were included.

To measure mean amplitudes, the peak amplitude of each of the ERP components was visually identified in the grand-mean waveforms, in each condition separately, at the electrode with greatest expected signal-to-noise ratio for each component based on previous literature (Näätänen and Picton, 1987; Friedman et al., 2001; Fonken et al., 2020). Thus, we used the Pz electrode to measure the P3b component, the Cz electrode for the P3a component, and the Cz electrode for the N1 component. The peak latency in the grand-averaged waveforms were used to obtain mean amplitudes for statistical comparison. Mean amplitudes were calculated using a 50 ms interval centered on the grand-mean peak, for each ERP component, separately for each stimulus category and position, in each condition, for each individual participant.

### 2.6. Statistical analyses

For behavioral data (HR and RT), separate two-way repeated measures ANOVA with factors of category (speech/music/environment) and position (1–8) to determine effects of category switch and category repetition. For event-related potentials (N1/P3a/P3b), separate two-way repeated measures ANOVA with factors of category (speech/music/environment) and position (1–8) were calculated to determine effects of category switch and category repetition on the mean amplitude of the ERPs. In cases where data violated the assumption of sphericity, the Greenhouse–Geisser estimates of sphericity were used to correct the degrees of freedom. Corrected *p* values are reported. Tukey’s HSD for repeated measures was conducted on pairwise contrasts for *post hoc* analyses when the omnibus ANOVA was significant. Contrasts were reported as significantly different at *p* < 0.05. Effect sizes were computed and reported as partial eta squared (*η*^2^*_p_*). Statistical analyses were performed using Statistica 13.3 software (Tibco).

## 3. Results

### 3.1. Passive auditory condition. Task: watch a movie

#### 3.1.1. P3a component

**Figure 2:**
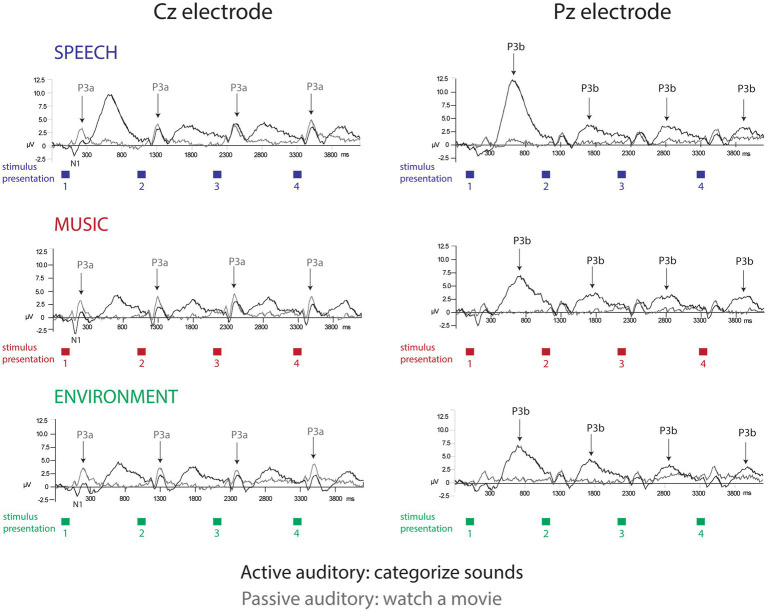
Event-related brain potentials. The grand-mean ERP waveforms elicited in each separate condition: active auditory (black traces) and passive auditory (gray traces) are displayed for the speech sounds (top row, blue), music sounds (middle row, red), and environmental sounds (bottom row, green). The Cz electrode (left panel) best displays the N1 and P3a components (labeled and with arrows for P3a). The Pz electrode (right panel) best displays the P3b component (labeled with arrows). The amplitude in microvolts is denoted along the *y*-axis. The colored squares displayed below the *x*-axis show the *stimulus presentation* rate, of one sound presented every 1.1 s. Categorical habituation and a speech processing bias are clearly demonstrated in the P3b amplitudes only during active task performance.

**Figure 3:**
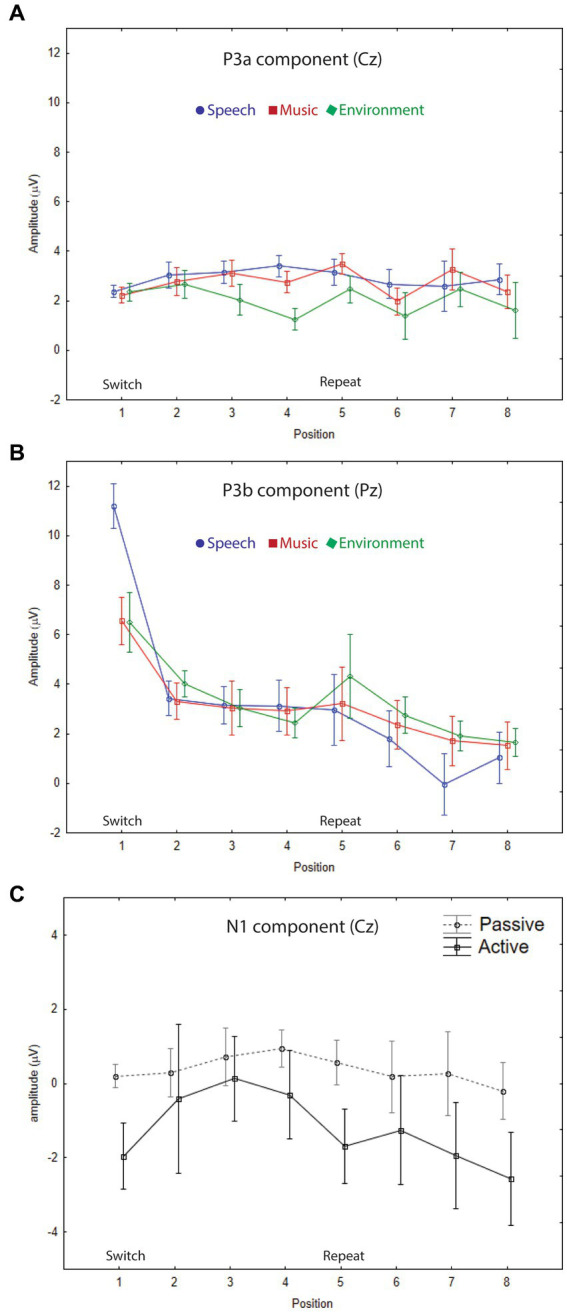
Grand-mean ERP amplitudes. **(A)** P3a component. The mean amplitudes and standard errors (whiskers) for speech (blue circle), music (red square), and environmental (green diamond) categories are overlain showing each stimulus position: switch (1–4) and repeat (5–8) trials, measured at the Cz electrode in the passive auditory condition. The onset of the switch and repeat trials are labeled along the *x*-axis, and the amplitude is denoted in microvolts along the *y*-axis for all panels. P3a amplitude did not index either category or expectation effects. **(B)** P3b component. The mean amplitudes and standard errors (whiskers) for speech (blue circle), music (red square), and environmental (green diamond) categories are overlain showing each stimulus position: switch (1–4) and repeat (5–8) trials, measured at the Pz electrode in the active auditory condition. A clear speech effect (larger amplitude for P3b speech in position 1), and a clear expectation effect (larger amplitude P3b for all categories at the switch (position 1)) are demonstrated. **(C)** N1 component. Grand-mean amplitudes and standard errors (whiskers) are compared for passive auditory (gray, dashed line) and active auditory (black, solid line) conditions at each stimulus position: switch (1–4) and repeat (5–8) trials, measured at the Cz electrode. Larger (more negative) N1 amplitudes were elicited by stimuli when a task was performed with the sounds.

**Figure 4:**
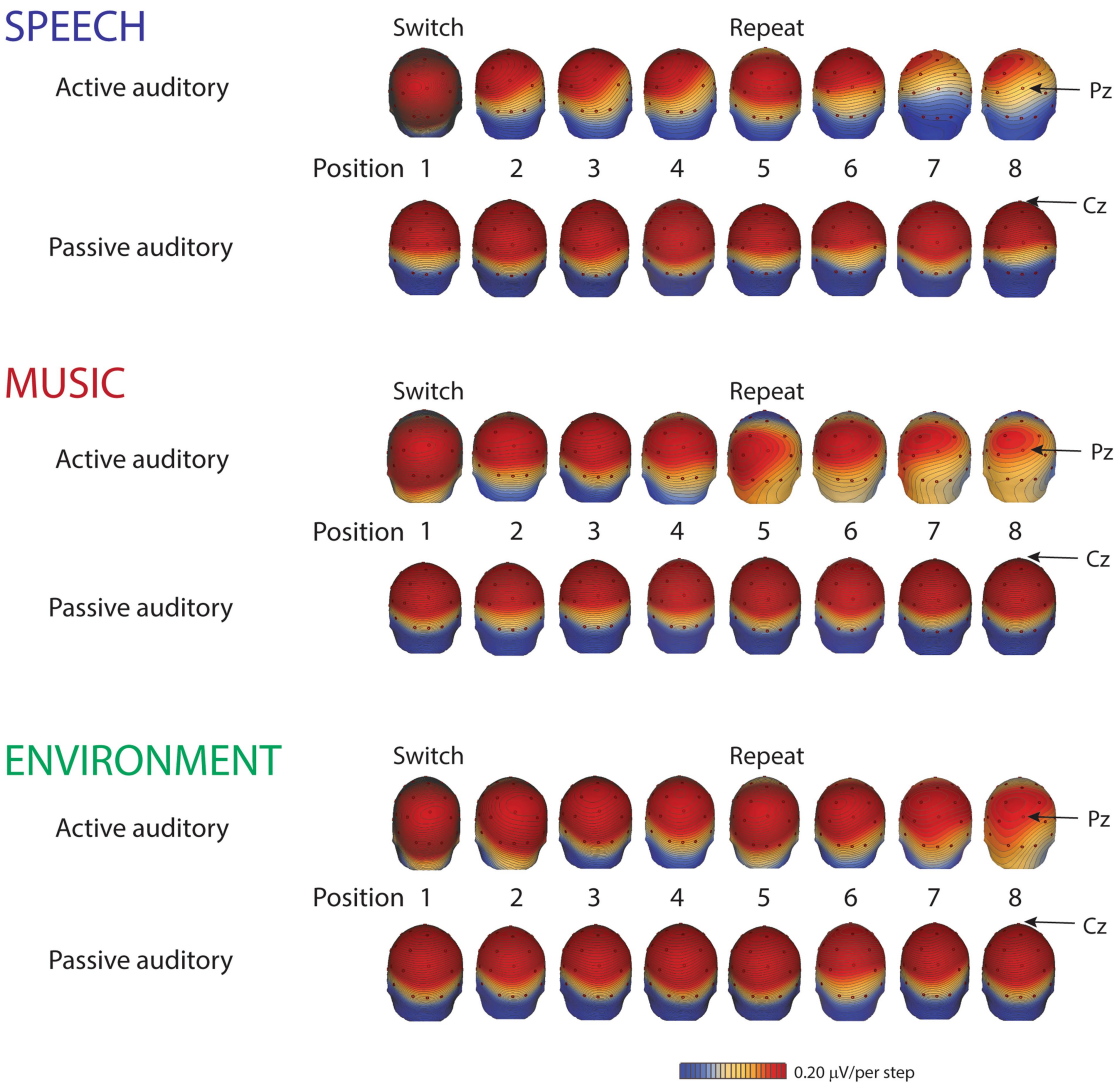
Scalp distribution maps. Scalp voltage distribution maps, from the grand-mean of all ten participants, show the P3b components (active auditory) and the P3a components (passive auditory), taken at their respective peak latencies, separately, by category for each position: speech (blue font), music (red font), and environment (green font). Small red dots denote each electrode. The Pz electrode, where the P3b amplitude is typically largest, is indicated with an arrow for the active auditory condition (top rows of each category). The Cz electrode, where the P3a amplitude is typically largest, is indicated with an arrow for the passive auditory condition (bottom rows of each category). Red indicates positive polarity; blue indicates negative polarity. The scale is 0.20 μV/step.

P3a amplitude did not differ as a function of position (*F*_7,63_ = 1.5, *p* = 0.25), or category (*F*_2,18_ = 2.0, *p* = 0.18), and there were no interactions (*F*_14,126_ = 1.4, *p* = 0.24). When the listener watched a movie, each sound engaged attention and elicited a P3a component with similar amplitudes across positions and sound categories (Figure 2, Cz electrode, left panel, gray solid line; Figure 3A; Figure 4, passive auditory). The salient categorical stimuli elicited an orienting response during both tasks (Figure 2, Cz electrode, left panel, compare gray and black traces).

### 3.2. Active auditory condition. Task: categorize the sounds

#### 3.2.1. P3b component

The P3b amplitude was larger (more positive) when elicited by the category switch stimulus for all three categories (position 1) compared to the category repetition stimuli (positions 2–8) (main effect of position, *F*_7,63_ = 10.33, *ε* = 0.24, *p* = 0.002, *η*^2^*_p_* = 0.53) (Figure 2, Pz electrode, right panel, black solid lines; Figure 3B; Figure 4, active auditory). This shows a dramatic decrease in the magnitude of the P3b amplitude after a single repetition of a categorical stimulus during active task categorization (compare the delta in peak P3b amplitude of responses to position 1 and 2 stimuli in Figure 2, right panel, Pz electrode, black traces, and Figure 3B). *Post hoc* analyses showed that there were no mean amplitude differences in responses elicited by stimuli in positions 2–8. The P3b amplitude remained attenuated for within-category repetitions, stimulus positions 2–4 after switching to a new category, and in stimulus positions 5–8 after repeating a category. The reduced P3b amplitude to category repeats in positions 2–8 demonstrates conceptual category “repetition suppression” during active identification (Figure 2, Pz electrode, right panel, black traces) that cannot be explained by stimulus-specific repetition suppression.

There was no main effect of category (*F*_2,18_ < 1, *p* = 0.83). However, there was an interaction between category and position (*F*_14,126_ = 3.7, *ε* = 0.27, *p* = 0.015, *η*^2^*_p_* = 0.29). *Post hoc* analyses revealed that P3b amplitudes elicited by position 1 stimuli were larger than positions 2–8 for all categories, and that the P3b amplitude elicited by spoken words in position 1 was larger than the P3b response to music and environmental sounds elicited in position 1 (with no amplitude difference between music and environment for position 1) (Figure 2, black traces, Pz electrode; Figure 3B). These results demonstrate both category habituation (position 1 larger than position 2 for all categories) and a speech effect (position 1 larger for speech than position 1 for music and environmental stimuli).

### 3.3. Sensory-specific processes

#### 3.3.1. N1 component

The sensory-specific N1 component, elicited during active and passive auditory tasks, did not clearly reflect categorical habituation or repetition suppression (Figure 2, Cz electrode, left panel, black and gray traces). There was a main effect of position (*F*_7,63_ = 4.8, *ε* = 0.49, *p* = 0.006, *η*^2^*_p_* = 0.35), with *post hoc* test showing that the N1 elicited in position 1 was more negative than the N1 in position 3 (but not with position 2) and position 1 did not differ in magnitude from any of the other N1 positions (Figure 2, Cz electrode). There was also a main effect of category (*F*_2,18_ = 56.6, *ε* = 0.98, *p* < 0.001, *η*^2^*_p_* = 0.42), with *post hoc* analyses showing that the N1 elicited by the music stimuli was larger in magnitude than either speech or environment. There was an attention effect, reflecting an expected attentional gain when attending vs. ignoring sounds (Hillyard et al., 1998). The N1 amplitude was larger (more negative amplitude) when the sounds were attended (main effect of attention, *F*_1,9_ = 5.5, *p* = 0.04, *η*^2^*_p_* = 0.38) (Figure 2, Cz electrode, left panel, compare gray and black traces; Figure 3C).

### 3.4. Task performance: categorizing the sounds

#### 3.4.1. Behavioral results

**Figure 5:**
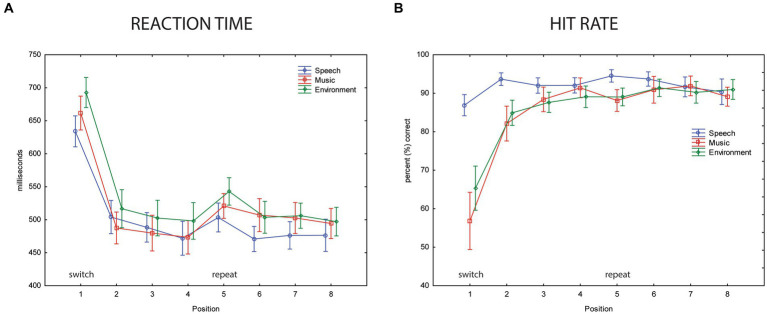
Behavioral data. **(A)** Reaction time. The mean reaction time (in ms, *y*-axis) for the three categorical sounds are overlain and displayed separately for each position (represented along the *x*-axis) for speech sounds (blue circle), music sounds (red square), and environmental sounds (green diamond). Position 1 is a category switch (labeled) and position 5 is a category repeat (labeled). Whiskers show the standard error. The slower mean reaction times in position 1 show a clear switch effect (implicit learning). **(B)** Hit rate. The mean hit rate in percentage (*y*-axis) is displayed for speech sounds (blue circle), music sounds (red square), and environmental sounds (green diamond) for each position (represented along the *x*-axis). Position 1 is a category switch and position 5 is a category repeat. Whiskers show standard error. A speech effect is seen in the lower mean hit rates to music and environmental sounds compared to speech in positions 1 and 2.

Performance results indicate implicit learning, with overall performance poorer when a category switch occurred (position 1, Figure 5). Mean reaction time was longest at the category switch (position 1, Figure 5A) (main effect of position, *F*_7,63_ = 29.32, *ε* = 0.32, *p* < 0.0001, *η*^2^*_p_* = 0.77). The switch stimulus (position 1) also had the lowest hit rates (main effect of position, *F*_7,63_ = 23.47, *ε* = 0.19, *p* < 0.0001, *η*^2^*_p_* = 0.72) (Figure 5B). The longer RT and lower HR may reflect the expectation of a category switch, in that additional processing would be at-the-ready to ‘re-identify’ the category after four sounds (i.e., in position 1). Once the category was identified, confirmation of category membership for stimuli 2–4 would only be needed, reflected by the faster RT and higher HR in positions 2–4 stimuli. Implicit learning is indicated by RT, which was, on average, 150 ms shorter to the second token of the within-category repetition (main effect of position: *F*_7,63_ = 29.3, *ε* = 0.32, *p* < 0.001, *η*^2^*_p_* = 0.77). *Post hoc* tests show that RT was slowest for position 1 stimuli. The faster responses time occurred for all within-category stimulus repetitions (positions 2–8). After only one repetition of a categorical stimulus, there was a dramatic decrease in RT (Figure 5A, compare positions 1 and 2).

For categorical effects, mean RT was shorter to speech and music sounds than to environmental sounds (main effect of stimulus category, *F*_2,18_ = 11.01, *ε* = 0.72, *p* = 0.003, *η*^2^*_p_* = 0.55). *Post hoc* calculations revealed the fastest reaction time to speech sounds, but not faster than music sounds when RT was collapsed across position (*p* = 0.13) (Figure 5A). The main effect of category on HR (*F*_2,18_ = 3.89, *ε* = 0.78, *p* = 0.039, *η*^2^*_p_* = 0.31) was due to a higher HR to speech than music sounds (*p* = 0.04) and HR for speech trended toward being higher than environmental sounds (*p* = 0.1). The significant interaction between sound category and position (*F*_14,126_ = 11.38, *ε* = 0.22, *p* < 0.0001, *η*^2^*_p_* = 0.56) was due to a higher HR for speech than music and environmental sounds at positions 1 and 2, whereas HR was not different across any positions for the speech sounds (Figure 5B). There was a significant interaction between sound category and position (*F*_14,126_ = 2.79, *ε* = 0.26, *p* < 0.05, *η*^2^*_p_* = 0.24). *Post hoc* calculations showing that in addition to a longer RT at position 1 across all sound categories, mean RT was longer in the repeat position 5 compared to position 4 for music and environmental sounds, but not for speech sounds. RT was not different between positions 4 and 5 for the speech sounds. There was an interaction between category and position (*F*_2,18_ = 11.0, *ε* = 0.26, *p* = 0.048, *η*^2^*_p_* = 0.55), which was due to slower RT in position 5 for music and environment. This may suggest anticipation of a switch in position 5 but no enhancement for speech, which already had faster response times.

## 4. Discussion

The key finding of our study, using a unique category repetition paradigm, was that extracting higher-level meaning from sound input requires specific task attention. This is the first study we know of showing effects of neural habituation for conceptual category repetition. We found three fundamental effects associated with actively categorizing sounds by speech, music, and environment: (1) categorical habituation; (2) implicit learning; and (3) a speech perception bias. None of these effects were observed when the stimulus sequences were presented, and the listener was watching a movie and had no specific task with the sounds. A crucial differentiating feature of our experimental design was that we dissociated stimulus repetition from category identification. Most previous studies that evaluate effects of predictive processing rely on repetition suppression where the same physical stimulus or pattern of stimuli are repeated. Higher-level conceptual effects can thus be conflated with stimulus-specific effects. In the current study, we uncoupled conceptual categorical effects from stimulus-specific effects by presenting categorical stimulus tokens that did not physically repeat. Using this experimental paradigm, the reduction of the P3b ERP amplitude that occurred at the first repetition of a category stimulus could not reflect stimulus-specific adaptation or ‘repetition suppression’. We found that categorical stimulus-repetition suppressed the brain response only when a task was being performed with the sounds. This suggests that category membership was only derived through task-based attentional processing.

### 4.1. Categorical habituation is a top-down phenomenon

Habituation is defined as a reduction in response to repeated stimulation that is not due to physiological effects, such as neural fatigue or adaptation (Magnussen and Kurtenbach, 1980; Rankin et al., 2009; Schmid et al., 2014). In the current study, we demonstrate a form of habituation that cannot be attributed to repetition suppression or neural fatigue. Conceptual categorical habituation was demonstrated by a dramatic decrease in the magnitude of the P3b amplitude after a single repetition of any of the categorical stimulus during active task categorization (Figures 2, 3). The delta in P3b amplitude from position 1 to position 2 was remarkable considering that the pattern of categorical stimuli occurred within an ongoing sequence of sounds, with no demarcation of when the grouping of category repetitions or switches were occurring. Further, the sounds themselves did not repeat, precluding stimulus-driven factors that could drive the response reduction by sensory adaptation or neural fatigue. The reduction in the magnitude of the neural signal after one repetition is notable because no two identical stimulus tokens were presented successively; the category repeated but not the physical stimuli. Accordingly, the reduction cannot be explained by stimulus-specific repetition suppression and reflects a neural habituation to the repetition of a conceptual category.

There was no categorical habituation when participants were watching a movie. It is well documented that when successive stimuli are acoustically unique, neural repetition suppression would not be expected (Näätänen and Picton, 1987; Budd et al., 1998; Grill-Spector et al., 2006). There was no conceptual “repetition suppression” when participants watched a movie. Thus, these results demonstrate that the conceptual categorical aspect of the stimuli was not automatically processed during passive listening. Habituation by repetition was only initiated when attention was focused on the sounds and a semantic categorical task was performed.

### 4.2. Evidence of implicit expectation only during active categorization of sounds

Expectation effects were observed only when the listener performed the categorization task with the sounds; not when they watched a movie. No explicit instructions were provided to participants about the stimulus structure, and the patterned structure was irrelevant to both tasks. However, implicit expectations could be formed by the regularity of the stimulus structure, in which the listener could expect a category switch after four successive categorical sounds most of the time. The larger P3b amplitude elicited by the categorical switch stimuli (position 1) demonstrates an implicit expectation that a category switch was likely to occur (a target switch). It should be noted that it was not possible to build up 100% expectation for a category switch because 30% of the time, rather than switching category, stimuli from the same category continued for a second successive group of four. Implicit category learning also influenced task performance. RT was slowest for position 1 stimuli: RT was 150 ms shorter to the second token of the within-category repetition. The faster responses time occurred for all the within-category stimulus repetitions (positions 2–8). The dramatic decrease in RT after only one repetition of a categorical stimulus is consistent with the substantial reduction in P3b amplitude after one categorical stimulus repetition (position 2) (Figure 3, compare positions 1 and 2). This is remarkable when considering that reaction time is faster to a repeated event than to a non-repeated event (Smith, 1968); that is, when it is the same physical stimulus. Here we show a reduction in RT to a conceptual repetition. The reduced response for position 2 stimuli indicates that the pattern of category repetition in the structure of the sequence was implicitly learned while performing the task. Implicit learning led to the knowledge that the category of stimuli would repeat after the switch position, even though that it was not the same physical sound token. The slower RT in position 1 and faster RT for positions 2–8 is consistent with modulation of the P3b amplitude, which was smaller after one category repetition and remained at the small amplitude until the next category switch. There was also an indication of implicit expectation in the longer RT at position 5, where a category switch may have been expected. However, this was not significantly reflected in the ERPs, likely due to the lower probability of a repeat than a switch.

In contrast, there was no evidence of implicit learning associated with the category switch when the listener watched a movie. The P3a amplitude did not differ as a function of position or category. The amplitude in position 1 was no different than that in any other position. Thus, a robust P3a was elicited by each successive stimulus token, with no indication by change in its magnitude that the brain detected a pattern of conceptual category repetitions. There was no categorical “repetition suppression.” Finding no index of implicit expectation, diverges from previous studies that have shown that stimulus repetition can build strong expectations and influence the brain response without attention focused on the sounds (Todorovic et al., 2011). However, our stimulus design is unique and may explain the differences in our results. The current study design differs from previous studies in two important ways. The repetition pattern of four sound tokens from the same category (speech, music, or environment) was comprised of four unique sound tokens from the category. For example, the listener may have heard the spoken words “peace” – “hello” – “yes” – “wonder” as the four-token repetition for one group in the speech category. All the sounds were different from each other. Therefore, identification of category repetition could not occur based on stimulus-driven features or acoustic characteristics of the sounds (Moskowitz et al., 2020). Secondly, expectations were not 100% predictable, that is, the category switch after the presentation of four sounds was not fully predictable; 30% of the time the category repeated. Consequently, during the passive condition, while attention was focused on reading captions and watching a movie, there could be some uncertainty about the regularity of the categorical aspect in the stimulus sequence, especially because the stimulus tokens themselves were not repeated, and attention was not actively monitoring the structure of the sound presentation. In addition, the structure of the sound sequence was irrelevant to performing the task. Thus, we conclude that attention focused onto the sounds with the intention to identify category membership was a key factor enabling expectations to be implicitly derived from the stimulus sequence.

### 4.3. Speech effects were observed only when attention was focused on the sounds

A surprising result of the study was that a speech bias was observed only during active listening. Response times were faster and ERP amplitudes were larger to speech category tokens during task performance. There was no categorical effect when listeners were passively listening and watching a movie. The automatic involuntary orienting response (indexed by the P3a component) did not differentiate speech from the other categories at any position (Figure 2, Cz electrode, gray traces), whereas the P3b amplitude did differentiate speech (Figure 2, Pz electrode, black traces). Moreover, there was an attentional orienting response to the sounds in both the active auditory and the passive auditory conditions (Figure 2, Cz electrode, left panel, compare black and gray traces). However, with the active auditory task, there was an additional P3b component elicited consistent with target detection. There was no P3b elicited in the passive auditory when there was no auditory task. Thus, only with attention focused on a task with the sounds, was there evidence that the higher-level categorical aspects of signal differentiation. That is, differentiation of the speech signal from other music and environmental sounds was only evident when attention was used to categorize the sounds. This is notable because there is considerable evidence from infancy showing that speech is processed differently from other environmental sounds (Eimas et al., 1971; Pisoni, 1979; Murray et al., 2006; Vouloumanos and Werker, 2007; Agus et al., 2012; Gervain and Geffen, 2019). Recent evidence, however, has suggested that speech may only show an ‘advantage’ under specific listening or task situations (Moskowitz et al., 2020). In previous studies showing a speech bias, this issue of attention may have not come to light because stimulus categories were not separated by unique tokens. Certainly, one can detect speech passively and unattended speech can alert our attention (e.g., the sound of your name being called) (Navon et al., 1987). However, the current results indicate that when attention is not directed towards the sounds, the acoustic characteristics that distinguish speech from other environmental sounds are not automatically discriminated as a special category when there is a complex mixture of sound categories occurring. Our results indicate that attentional control is required to process the higher-level aspects of the speech signal, to extract the conceptual category (speech, music, or environment) when there are a variety of complex sounds. Speech may not be treated as a distinct or separate category without an active task and attention to the sounds. A question that remains is how specific the task must be to the conceptual process for it to alter the neural response; would performing a task not involving categorization also show no category effects?

## 5. Summary and conclusions

Our results address a fundamental controversy about the role of attention in higher level processing. We distinguished between repetition suppression and conceptual categorical habituation by repeating sounds that fit a sound category but never repeating the same physical sound tokens. Predictive processing theory suggests that brain processes are continually generating and updating a model of the environment (Winkler et al., 1996; Friston, 2005). This theory suggests that the brain automatically builds expectations (priors), derived by sound patterns extracted through stimulus statistics. Thus, our results diverge somewhat from this aspect of the predictive processing theory in that we found no reduction in the magnitude of the neural response to a repeated sound category unless attention was directed to the categorical aspect of the sounds. The theoretical perspective that the brain calculates and anticipates all stimulus patterns within a sound sequence and automatically sets up expectations, implicitly learned without attention, is not upheld for higher-level conceptual categories involving a mixture of complex sounds with the current experimental design. We found no evidence of implicit learning of the structured sound sequence when the listener was passively listening and watching a movie. Our results are consistent with previous studies showing that task goals, rather than stimulus statistics, have great influence on neural processing of auditory and visual patterns (Sussman et al., 1998, 2002; Max et al., 2015; Solomon et al., 2021).

Overall, we found that top-down knowledge was required to set up expectations for higher-level processes (Rao and Ballard, 1999; Summerfield et al., 2008). Our results thus link in with the question of how much, or what type of, processing of the unattended, irrelevant sounds occurs when performing another task. It is generally thought that attention can ‘leak’ or ‘slip’ to the unattended stimuli while performing another task (Lachter et al., 2004). Watching a movie is not considered a highly demanding task, and it may be argued that attentional slips could easily occur. However, remarkably, there was no evidence of implicit learning of the structured sound sequence, or of categorical perception, such as a speech bias during passive listening, when it would be more likely there would have been potential slips of attention to the unattended sounds. These findings are consistent with the theory of Broadbent (1956), who proposed that attention is a limited resource and therefore attention to one set of sounds limits available resources to process the unattended sounds, beyond the simple sound features (e.g., frequency, intensity, spatial location). We suggest that the limited resource here is higher-level conceptual category formation. We found that the ‘slippage’ of attention to irrelevant sounds was not enough to induce higher-level processing, indicating that those higher-level processes that identify linguistic, semantic, or categorical aspects of stimuli require some form of active attention. Although humans are experts at detecting and finding patterns in sensory input, the extent of processing and the role of attention in processing irrelevant sounds, under various listening situations, is still yet to be fully resolved.

## Data availability statement

The raw data supporting the conclusions of this article will be made available by the authors, without undue reservation.

## Ethics statement

The studies involving humans were approved by Albert Einstein College of Medicine. The studies were conducted in accordance with the local legislation and institutional requirements. The participants provided their written informed consent to participate in this study.

## Author contributions

HM: conceptualization, methodology, formal analysis, writing – original draft, and visualization. ES: conceptualization, methodology, formal analysis, writing – review and editing, visualization, supervision, and funding acquisition. All authors contributed to the article and approved the submitted version.
