# Supplementary material for: Sound category habituation requires task-relevant attention
Source: Front Neurosci. 2023 Oct 24;17:1228506. doi: 10.3389/fnins.2023.1228506 (PMC10628171; doi:10.3389/fnins.2023.1228506)
Supplement: Supplementary file 1 [file Data_Sheet_1.PDF]

APPENDIX I. List of sounds by category

**SPOKEN WORDS**	**MUSICAL SOUNDS**	**ENVIRONMENTAL SOUNDS**	**ENVIRONMENTAL SOUNDS**
“and”	accordion	airplane	paper tearing
“beautiful”	bagpipe	ball bounce	phone
“echo”	banjo	bicycle bell	rain
“eye”	bass	bird chirping	sandpaper
“faster”	bells	boat horn	sea lion
“female”	brass	brush teeth	sheep
“focus”	cello	camera	siren 1
“form”	clarinet	can pop top	siren 2
“freedom”	cymbals	car crash	thunder2
“gentlemen”	drumroll	car honk1	ticking clock
“goodbye”	flute	car honk2	toilet
“healthy”	gong	cat meow	train
“hello”	guitar	chicken	train horn 1
“love”	harmonica	cow moo	train horn 2
“mom”	harp	cricket	train horn 3
“muse”	keyboard	crow	trolley
“no”	organ	cut paper	truck
“okay”	piano	dog bark	typewriter
“peace”	recorder	donkey	water 1
“please”	saxophone	doorbell	water 2
“rich”	snare drum	duck	water 3
“two”	trumpet	frog	water drip
“why”	viola	knocking	wind 1
“yanked”	violin	lion	wind 2
“yes”	xylophone	monkey	wind 3
		mosquito	wind 4
		motorcycle	windchime
		ocean	zipper
		owl	
